# Chemical and Antioxidant Properties of Wild Edible Mushrooms from Native *Nothofagus* spp. Forest, Argentina

**DOI:** 10.3390/molecules21091201

**Published:** 2016-09-08

**Authors:** Carolina V. Toledo, Carolina Barroetaveña, Ângela Fernandes, Lillian Barros, Isabel C. F. R. Ferreira

**Affiliations:** 1 Centro de Investigación y Extensión Forestal Andino Patagónico (CIEFAP), Ruta 259, Km 4, Esquel 9200, Chubut, Argentina; ctoledo@correociefap.org.ar; 2 Facultad de Ingeniería, Universidad Nacional de la Patagonia S.J. Bosco, Ruta 259, Km 4, Esquel 9200, Chubut, Argentina; 3 Consejo Nacional de Investigaciones Científicas y Tecnológicas (CONICET), Av. Rivadavia 1917, C1033AAJ, CABA, Argentina; 4 MountainResearch Centre (CIMO), ESA, Polytechnic Institute of Bragança, Campus de Santa Apolónia, 1172, 5300-253 Bragança, Portugal; afeitor@ipb.pt (A.F.); lillian@ipb.pt (L.B.); 5 Laboratory of Separation and Reaction Engineering–Laboratory of Catalysis and Materials (LSRE-LCM), Polytechnic Institute of Bragança, Campus de Santa Apolónia, 1134, 5301-857 Bragança, Portugal

**Keywords:** macrofungi, nutrients, nutritional value, phenolic compounds, antioxidant

## Abstract

This study addresses issues regarding chemical and bioactive properties of nine wild edible mushrooms from native *Nothofagus* forest from Patagonia, Argentina. Macronutrients, sugars, fatty acids, tocopherols, organic acids, phenolic compounds and antioxidant properties were determined. Protein was found in high levels and varied between 3.35 g/100 g dw in *Cyttaria hariotii* and 22.29 g/100 g dw in *Lepista nuda*. All of them presented mannitol and trehalose as main sugars. Mannitol was significantly higher in *Ramaria patagonica*, although absent in *Fistulina endoxantha*, whereas trehalose predominated in *Aleurodiscus vitellinus*, *Hydropus dusenii*, *Cortinarius magellanicus*, *C. hariotii*, *Grifola gargal* and *L. nuda*, ranging from 1.15 to 10.26 g/100 g dw; it was absent in *R. patagonica*. The major fatty acid found was linoleic acid, followed by oleic acid and palmitic acid. All species presented oxalic and fumaric acids, while some also had malic, quinic and citric acids. Tocopherols composition was variable. *Cortinarius magellanicus* presented significantly higher contents of both α-tocopherol and β-tocopherol. *R. patagonica* presented the best results in all the antioxidant activity assays (EC_50_ values ≤ 1 mg/mL) and the highest content of phenolic compounds presenting gallic, *p*-hydroxybenzoic, *p*-coumaric and cinnamic acids. This study constitutes the first report on chemical composition and nutritional value of most of these edible mushroom species. Furthermore, it provides important information necessary to characterize and define the use of these species as gastronomic delicacies, functional foods and sources of bioactive compounds.

## 1. Introduction

The consumption of wild edible mushrooms in cooking, traditional medicine and religious rituals is a practice that dates back to the beginnings of civilization [1] and has been developed and maintained in different countries around the world, particularly China in Asia [2,3,4], Spain and Italy in Europe [1] and Mexico in Latin America [5].

Wild mushrooms are becoming more and more important in our diet for their nutritional value, including high protein, vitamins and minerals and low fat/energy contents [6,7,8,9]. The fatty acids composition may also have beneficial effects on blood lipid profiles. Substitution of saturated fatty acids (SFAs) with monounsaturated fatty acids (MUFAs) leads to increased high density lipoprotein (HDL) cholesterol and decreased low-density lipoprotein (LDL) cholesterol, triacylglycerols, lipid oxidation and LDL susceptibility to oxidation [10]. The study of edible fungi in various parts of the world has included the analysis of the chemical composition and nutritional values as elements necessary to characterize and define other uses besides the gastronomic, as reported for wild mushrooms and cultivated in Spain [11], Italy [12], Portugal [13,14], Greece [15,16], Turkey [17], India [18], Iran [19], Thailand [20], Serbia [21], Brazil [22], Mexico [23] and Argentine [24,25,26]. In recent years, increased interest in human health, nutrition and disease prevention has enlarged consumer demand for functional foods [27,28,29]. In fact, mushrooms have become attractive as functional foods and as a source of bioactive compounds [30,31]. Some species are rich sources of antioxidant compounds such as phenolic compounds and tocopherols [32,33,34,35,36,37,38].

*Nothofagus* spp. forests (in Patagonian Andes region, Argentina) harbor several fungal species [39,40] with edible potential and high nutritional value. In particular, the species *Aleurodiscus vitellinus* (Lev.) Pat., *Cortinarius magellanicus* Speg., *Hydropus dusenii* (Bres.) Singer., *Cyttaria hariotii* E. Fisch. *Fistulina antarctica* Speg., *F*. *endoxantha* Speg., *Grifola gargal* Singer, *Lepista nuda* (Bull.) Cooke. and *Ramaria patagonica* (Speg.) Corner, have records of consumption. However, background information on their chemical composition and bioactive properties is very scarce and restricted to *C. hariotii* [41,42], *G. gargal* [43,44,45,46,47,48] and *L. nuda* [13,15,49,50].

Knowledge on chemical composition allows the use of wild fungi as food or bioactive resources and favors regional economies related to non-wood forest products harvesting, gastronomy and health care. Diverse uses of these resources would promote better management and conservation actions on the habitat where they grow.

This study addresses issues regarding chemical and bioactive properties of wild edible mushrooms from native *Nothofagus* forest, in order to evaluate them as sources of nutrients and bioactive compounds. Chemical analysis included determination of macronutrients and individual profiles in sugars, fatty acids and organic acids. The bioactivity evaluation was focused on the analysis of phenolic compounds (total and individuals) and antioxidant properties (free radicals scavenging activity, reducing power and inhibition of lipid peroxidation).

## 2. Results and Discussion

### 2.1. Nutritional Value

The results of the composition in terms of nutrients and estimated energetic value (expressed in dry weight basis) obtained for the nine wild edible fungal species are shown in Table 1.

Protein was found in high levels and varied between 3.35 g/100 g dw in *C. hariotii* and 22.29 g/100 g dw in *L. nuda*. The highest protein content observed in *L. nuda* was within the range of previous studies, that reported values between 19.8 and 59.4 g/100 g dw [13,15,49]. Comparing *R. patagonica* with other Ramaria species, *R. flava* gave higher values (35.55 g/100 g dw) [51], as also *R. botrytis* (39.8 g/100 g dw) [13]. *C. magellanicus* and *H. duseni*, within a range of 13 to 16 g/100 g dw of protein content, showed similar values to those reported for *Suillus granulatus* (L.) Roussel (16.5 g/100 g dw) and *Cortinarius praestans* Cordier (14.54 g/100 g dw) by Ouzouni and Riganakos [15] and Pereira et al. [52], respectively. *G. gargal*, *A*. *vitellinus* and *F*. *endoxantha* yielded values between 5 to 8 g/100 g dw. Previous studies on *G. gargal* reported similar results to those found in this one [43]. *F. antarctica* and *C. hariotii* showed the lowest values, between 3 to 4 g/100 g dw protein content, the former showing lower values (7.50 g/100 g dw) to those obtained by Schmeda-Hirschmann et al. [42]. Other studies with different species of Fistulina have reported higher values (63.69 g/100 g) [53] than those reported here for *F. antarctica* and *F. endoxantha*. Protein content of mushroom species could be affected by a number of factors, namely the developmental stage, the sampled part, the level of available nitrogen and location [54]. Furthermore, the conversion factor used to calculate the protein content can influence its outcome [8,55].

Crude fat content ranged from 0.83 g/100 g dw in *F. antarctica* and 4.29 g/100 g dw in *H. dusenii*. Previous reports of *C. hariotii* and *G. gargal* fat content were higher than those reported here (5 and 7.5 g/100 g dw, respectively) [42,43]. *L. nuda* fat content was close to that obtained by Colak et al. [49] for the same species (9.04 g/100 g dw). Carbohydrates were also an abundant macronutrient and ranging from 67.58 g/100 g dw in *C. magellanicus* to 94.22 g/100 g dw in *F. antarctica*. Comparisons with previous reports showed lower values for *C. hariotii* (75 g/100 g dw) [42] and similar for *G. gargal* [43] and *L. nuda* [15].

Ash content was also variable between species and ranged from 1.24 g/100 g in *F. antarctica* to 13.92 g/100 g in *C. magellanicus*. The parasitic *C. hariotii* yielded ash values lower (7.0 g/100 g dw) than what have been previously reported by Schmeda-Hirschmann et al. [42], whereas *L. nuda* showed higher values (5.4 and 6.0 g/100 g dw) than those reported by Colak et al. [49] and Ouzouni & Riganakos [15] respectively and lower values (18.5 g/100 g dw) than the reported by Barros et al. [13]. *G*. *gargal* yielded lower values than what reported by Schmeda-Hirschmann et al. [43] for the same species (5.0 g/100 g dw).

The highest value of energetic contribution was observed in *F. antarctica*, while *C. magellanicus* presented the lowest value (Table 1). The energy contribution of the different species under study was calculated on the basis of nutritional analysis (Table 1); the highest values were observed in *F*. *antarctica* (399.18 kcal/100 g dw), while *C. magellanicus* presented the lowest value (358.03 kcal/100 g dw).

Concerning sugars composition (Table 2), the nine mushrooms showed some homogeneity. All of them presented mannitol and trehalose as main sugars, as has been previously reported for other wild mushroom species [13,35,52]. Mannitol content was significantly higher for *R. patagonica*, although absent in *F. endoxantha*, whereas trehalose predominated in *A. vitellinus*, *H. dusenii*, *C. magellanicus*, *C. hariotii*, *G. gargal* and *L. nuda*, ranging from 1.15 to 10.26 g/100 g dw and was absent in *R. patagonica*. Other sugars such as fructose and rhamnose were present in lower abundance in some of the studied species, but predominated in *F. endoxantha* and *F. antarctica*, respectively. Sucrose was only detected in *H. dusenii.* In terms of total sugars content, *F*. *antarctica* revealed the highest value (28.68 g/100 g dw), while *C. hariotii* the lowest (4.02 g/100 g dw). This is the first report of sugar profiles for the species included in this study, except *L. nuda*, which has been described by Colak et al. [13,15,49]. *A*. *vitellinus* and *C*. *hariotii* presented similar quantities of trehalose than *Agaricus lutosus* (Møller) Møller and *Leucoagaricus leucothites* (Vittad.) Wasser, respectively [52]. Meanwhile, mannitol values obtained for *H. dusenii* and *R. patagonica* were similar to the ones reported by Barros et al. [35] for *Cantharellus cibarius* L. ex Fr. and *Marasmius oreades* (Bolt. ex Fr.) Fr., respectively. Mannitol and trehalose concentrations on dry specimens of *L*. *nuda* were different (0.80 g/100 g and 12.03 g/100 g dw, respectively) from those reported by Barros et al. [35].

The results of fatty acids composition, total saturated fatty acids (SFA), monounsaturated fatty acids (MUFA) and polyunsaturated fatty acids (PUFA) of the studied mushrooms are shown in Table 3. In general, the major fatty acid found in the studied species was linoleic acid (C18:2), followed by oleic acid (C18:1) and palmitic acid (C16:0). Besides the three main fatty acids already described, 21 more were identified and quantified. PUFA were the main group of fatty acids, significantly higher than MUFA and SFA in *H. dusenii*, *F. antarctica*, *F. endoxantha*, *R. patagonica*, *C. hariotii*, *C. magellanicus* and *L. nuda*, the latter with the highest value due to the high contribution of linoleic acid (72%). In *A. vitellinus* and *G. gargal*, MUFA were the main group, due to the high contribution of oleic acid, ranging from 45% to 52%. UFA (unsaturated fatty acids) predominated over SFA in all the studied species, ranging from 64.1% and 83.40%. The main fatty acids found in this study were linoleic acid, followed by oleic acid and palmitic acid, coincidently with that observed for species of mushrooms at different studied here [52,56,57,58,59]. Oleic acid is a monounsaturated fatty acid omega 9 series typical of vegetable oils such as olive oil and is known for its effectiveness in reducing cholesterol levels [60], which promotes the decrease of cardiovascular diseases [61]. Linoleic acid is an essential fatty acid as it cannot be synthesized by the human organism, due to the lack of desaturase enzymes required for its production [62].

Regarding the organic acids composition, all species presented oxalic and fumaric acids; some species also revealed the presence of malic, quinic and citric acids (Table 4). The oxalic acid content revealed significant differences among species, being higher for *L. nuda* and *R. patagonica* although most species showed lower concentrations, between 0.10 to 0.54 g/100 g dw. Fumaric acid, with antioxidant, antimicrobial and acidifying properties [63], was higher for *C. magellanicus*, while *A. vitellinus* and *C. hariotti* presented the lowest values. Malic acid was found in five of the nine studied species, *C. magellanicus* with the highest content and *C. hariotti* with the lowest content. Quinic acid was only detected in *F. antarctica* and *L. nuda* while citric acid, important in the prevention of mushrooms browning [63] was only present in *C. magellanicus* and *R. patagonica*. The total organic acids content ranged from 0.21 g/100 g dw in *A. vitellinus* to 28.87 g/100 g dw in *L. nuda*. This is the first report on organic acids composition for all the studied species, except *L. nuda*, whose previous reported values were higher (4.34 g/100 g) [64] than those found in this work.

Tocopherol composition differed between the studied species (Table 5). *C. magellanicus* presented significantly higher contents from both α-tocopherol and β-tocopherol than the other species. The β-tocopherol was also present in significantly decreasing values in *L*. *nuda*, *F*. *endoxantha* and *G*. *gargal*. The α-tocopherol, present in all species that presented tocopherols, showed minor proportions than β-tocopherol. Previous reports of tocopherols of *L*. *nuda* were detected and quantified, but using different methodologies and other units [13,34,59]. This work represents the first contribution in relation to the detection of tocopherols for the other eight studied species.

### 2.2. Bioactive Compounds and Antioxidant Properties

Three phenolic acids (galic, *p*-hydroxybenzoic and *p*-coumaric acids) and a related compound (cinnamic acid) were identified and quantified in some of the studied species (Table 6). Gallic acid was detected in a range between 1.26 and 4.56 µg/100g dw, but was absent in *H. dusenii*, *C. magellanicus* and *L. nuda*. The lowest values of gallic acid were found in *A. vitellinus*, *C. hariotii* and *G. gargal* without statistical differences (*p* < 0.05). The *p*-hydroxybenzoic acid was only present in *F*. *antarctica* and *R*. *patagonica*, while *p*-coumaric acid was registered only in *R. patagonica*. This last species showed a noticeably higher phenolic acid concentration, mostly due to the contribution of *p*-hydroxybenzoic acid, compared with the other species. Barros et al. [50] working with Portuguese wild mushrooms also detected other species of the genus *Ramaria* (*R. botritys*) as the one with the highest phenolic acids concentration (356.7 mg/kg, dry matter). *L. nuda* did not show phenolic compounds at all in this study, although Barros et al. [50] reported the presence of *p*-hydroxybenzoic acid, *p*-coumaric acid and protocatechuic acid. These differences could be related to the mushroom origin and the edaphoclimatic conditions as well as stress conditions such as UV radiation, infection by pathogens and parasites, wounding, air pollution and exposure to extreme temperatures [50]. This is the first report concerning the phenolic acid composition of the other eight studied species.

The composition in total phenolics and in vitro antioxidant activity of the studied wild mushrooms is shown in Table 7. *R. patagonica* presented the best results in all the antioxidant activity assays, with EC_50_ values of DPPH scavenging activity, reducing power, β-carotene bleaching inhibition and TBARS inhibition ≤ 1 mg/mL, in agreement with its highest levels of total phenolics. In fact, it had been reported that the antioxidant activity of mushroom materials is well correlated with the content of phenolic compounds [57]. *F. antarctica* showed the lowest antioxidant activity (EC_50_ values ranging from 0.95 to 13.8 mg/mL) and low content of phenols (7.82 mg GAE/g extract), very close to those of *C. hariotii*, *G. gargal* and *C. magellanicus*.

The studied samples of *L. nuda* showed lower values of DPPH radical-scavenging activity, reducing power and β-carotene bleaching inhibition (16.05, 2.08 and 12.24 mg/mL, respectively) than those recorded by Pinto et al. [59], while TBARS inhibition value was similar (5.80 mg/mL) to that reported by Barros et al. [35]. Elmastas et al. [34] evaluate the antioxidant activity, *L*. *nuda* by applying a different methodology, while; Brujin et al. [44,45] did so for *G*. *gargal*, but using different solvents or heat treatment. Postemsky et al. [46], in a study of different species of *Grifola*. spp. on solid state fermentation to obtain biotransformed wheat grain, reported higher DPPH radical-scavenging values (0.55 mg/mL) and reducing power (0.56 mg/mL) activity for *G*. *gargal* than those detected in this work.

## 3. Materials and Methods

### 3.1. Collection and Identification of Fungi

**Figure 1:**
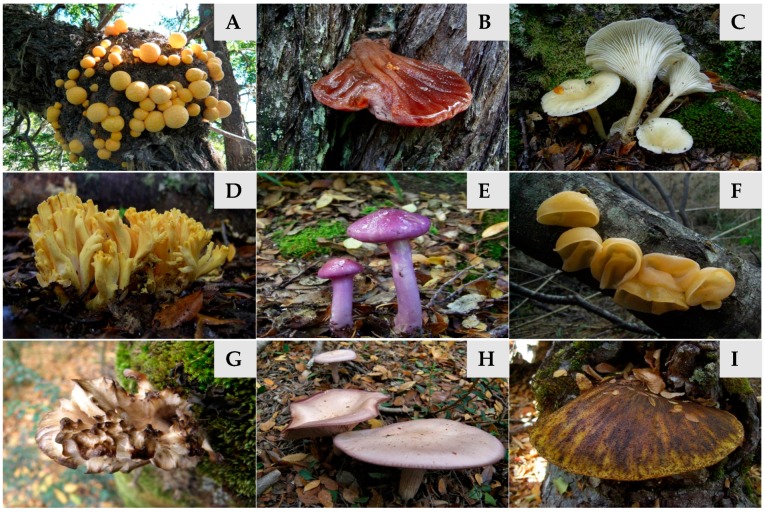
Frutifications of the wild studied mushrooms. (**A**) *Cyttaria hariotii*; (**B**) *Fistulina antarctica*; (**C**) *Hydropus dusenii*; (**D**) *Ramaria patagonica*; (**E**) *Cortinarius magellanicus*; (**F**) *Aleurodiscus vitellinus*; (**G**) *Grifola gargal*; (**H**) *Lepista nuda*; (**I**) *Fistulina endoxantha*.

Specimens of nine species of wild edible mushrooms (*Aleurodiscus vitellinus*, *Cortinarius magellanicus*, *Hydropus dusenii*, *Cyttaria hariotii*, *Fistulina antarctica*, *F. endoxantha*, *Grifola gargal*, *Lepista nuda* and *Ramaria patagonica*) (Figure 1) were collected during fall and spring seasons of 2010, 2011 and 2012 in *Nothofagus* forest from National Parks of Chubut, Río Negro and Neuquén provinces, Argentina. The morphological identification of the collected specimens was performed according to previous reports [65,66,67,68]. Representative collections of each species was herborized and deposited in the Herbarium of the Patagonian Forest Research Center (CIEFAP; Esquel, Chubut, Argentina). Individual samples were pulverized and stored in freezer at −18 °C in polyethylene bags for subsequent chemical analyses. Species analyses were performed on composite samples, including fruit bodies from collections from different stands.

### 3.2. Standards and Reagents

Acetonitrile (99.9%), *n*-hexane (97%) and ethyl acetate (99.8%) were of HPLC grade from Fisher Scientific (Lisbon, Portugal). The fatty acids methyl ester (FAME) reference standard mixture 37 (standard 47885-U) was purchased from Sigma (St. Louis, MO, USA), as also were other individual fatty acid isomers, l-ascorbic acid, trolox (6-hydroxy-2,5,7,8-tetramethylchroman-2-carboxylic acid), tocopherol, organic acid, phenolic acids and sugar standards. Racemic tocol, 50 mg/mL, was purchased from Matreya (Pleasant Gap, PA, USA). 2,2-Diphenyl-1-picrylhydrazyl (DPPH) was obtained from Alfa Aesar (Ward Hill, MA, USA). Water was treated in a Milli-Q water purification system (TGI Pure Water Systems, Greenville, SC, USA).

### 3.3. Determination of Nutritional and Chemical Composition

#### 3.3.1. Macronutrients

Mushroom samples were analyzed for chemical composition (protein, fat, carbohydrates and ash) using AOAC procedures [69]: Crude protein (AOAC 978.04), fat (AOAC 920.85), ash (AOAC 923.03) and total carbohydrates were calculated by difference. Results were expressed in g per 100 g of dry weight (dw).

Energy was calculated according to the following equation: Energy (Kcal) = 4 × (g proteins + g carbohydrates) + 9 × (g lipids) [12]. Results were expressed in kcal per 100 g of dry weight (dw). 

#### 3.3.2. Sugars Composition

Free sugars were determined by high performance liquid chromatography coupled to a refraction index detector (HPLC-RI) based on the method used by Harada et al. [70] with minor modifications optimized by Heleno et al. [53]. Sugars were identified by comparing the relative retention times of sample peaks with standards and data was processed using a Clarity 2.4 Software (DataApex, Podohradska, Czech Republic). Quantification was performed using an internal standard (IS, raffinose) methodology and results were expressed in g per 100 g of dry weight.

#### 3.3.3. Fatty acids Composition

Fatty acids (obtained after Soxhlet extraction) were determined by gas chromatography with flame ionization detection (GC-FID)/capillary column as described previously by the Heleno et al. [53] and after the following trans-esterification procedure. Fatty acids were processed using a Clarity 4.0.1.7 Software (DataApex, Podohradska, Czech Republic), identification was performed by comparing the relative retention times of FAME peaks from samples with standards and results were and expressed in relative percentage of each fatty acid.

#### 3.3.4. Organic Acids Composition

Organic acids were determined by ultra-fast liquid chromatography coupled to a photo diode array detector (UFLC-PDA) following a procedure previously described by the authors [64]. The identification was performed by comparing relative retention times and UV spectra with commercial standards. Quantification was accessed by area comparison of the peaks recorded at 215 nm with calibration curves obtained from commercial standards and results were expressed in g per 100 g of dry weight. 

#### 3.3.5. Tocopherol Composition

Tocopherol content was determined following a procedure previously optimized and described by Heleno et al. [36], using a HPLC system (Smartline, Knauer, Germany), coupled to a fluorescence detector (FP-2020; Jasco, Japan) programmed for excitation at 290 nm and emission at 330 nm. Data was processed using a Clarity 2.4 Software (DataApex). Compounds were identified by chromatographic comparisons with commercial standards and quantification was based on the fluorescence signal response, using an internal standard method. Tocopherol contents in mushrooms were expressed in µg per 100 g of dry mushroom.

### 3.4. Evaluation of Bioactive Compounds and Antioxidant Properties

#### 3.4.1. Extracts Preparation

The extractions were performed using a fine dried powder (20 mesh; 2 g) stirred with 30 mL of methanol at 25 °C at 150 rpm for 1 h and filtered through Whatman No. 4 paper. The residue was then re-extracted with one additional 30 mL portion of methanol. The combined methanolic extracts were evaporated at 35 °C under reduced pressure (rotary evaporator Büchi R-210). The extracts were re-dissolved in methanol to a final concentration 20 mg/mL and were further diluted to different concentrations to be submitted to the following assays.

#### 3.4.2. Phenolic Compounds Composition

*Total phenolics*. Phenolics were estimated based on procedures previously described by Wolfe et al. [71]. Gallic acid was used to calculate the standard curve (0.05–0.8 mM; Y = 1.8072x + 0.0211; *R*^2^ = 0.999) and results were expressed as mg of gallic acid equivalents (GAE) per g of extract.

*Individual phenolic compounds*. Phenolic compounds were determined by UFLC-PDA (Shimadzu Corporation, Kyoto, Japan) following a procedure previously described by the authors [72], with some modifications. The phenolic compounds were identified by comparing their retention time and UV-vis spectra with those obtained from commercial standards, when available. Quantification was obtained using calibration curves for each available phenolic standard and for the identified phenolic compounds for which a commercial standard was not available, quantification was based on calibration curves using compounds from the same phenolic group. Results were expressed in µg per 100 g of dry weight.

#### 3.4.3. Antioxidant Properties

The antioxidant activity was evaluated by DPPH radical-scavenging activity, reducing power, inhibition of β-carotene bleaching in the presence of linoleic acid radicals and inhibition of lipid peroxidation using TBARS in brain homogenates [7,57]. The extract concentrations providing 50% of antioxidant activity or 0.5 of absorbance (EC_50_) were calculated from the graphs of antioxidant activity percentages (DPPH, β-carotene bleaching and TBARS assays) or absorbance at 690 nm (reducing power assay) against extract concentrations. Trolox was used as standard.

#### 3.4.4. Statistical Analysis

For mushroom species, three independent samples were analyzed and data was expressed as mean ± standard deviation. All statistical tests were performed at a 5% significance level using IBM SPSS Statistics for Windows, version 22.0. (IBM Corp., Amonk, NY, USA). The fulfilment of the one-way ANOVA requirements, specifically the normal distribution of the residuals (data not shown) and the homogeneity of variance, was tested by means of the Shapiro Wilk’s and the Levene’s tests, respectively. All dependent variables were compared using Tukey’s honestly significant difference (HSD) or Tamhane’s T2 multiple comparison tests, when homoscedasticity was verified or not, respectively.

## 4. Conclusions

In conclusion, this study constitutes the first report on the chemical composition of *A. vitellinus*, *H. dusenii*, *C. magellanicus*, *F. antarctica*, *F. endoxantha* and *R. patagonica.* The studied species showed low fat values and high protein and carbohydrate contents, similar to what have been reported in previous studies involving wild edible mushrooms. Nevertheless, the high nutritional quality and unique organoleptic characteristics have to be documented in order to initiate the formal registration of many of these novel species in the Argentinian code of food. On the other hand, discovering new sources of nutraceuticals from these species put in value these non-timber forest products from Patagonia native forest and pose the challenge to work for a better management and conservation of this natural resource and habitats related to them. In particular, *R. patagonica* gave the highest antioxidant activity and the highest content of phenolic compounds.

## Figures and Tables

**Table 1:** Proximate chemical composition (g/100 g) and energetic value (kcal/100 g) of the studied wild mushrooms on a dry weight basis (mean ± SD).

Species	Total Fat	Crude Protein	Carbohydrates	Ash	Energy
*A. vitellinus*	3.49 ± 0.20b	7.38 ± 0.21e	84.23 ± 0.32d	4.90 ± 0.05e	397.84 ± 0.56ab
*H. dusenii*	4.29 ± 0.22a	13.52 ± 0.43d	75.96 ± 0.86g	6.23 ± 0.45d	396.53 ± 1.99abc
*C. magellanicus*	2.75 ± 0.12c	15.75 ± 0.10c	67.58 ± 0.27i	13.92 ± 0.17a	358.03 ± 0.08g
*C. hariotii*	1.31 ± 0.03de	3.35 ± 0.15g	92.51 ± 0.25b	2.83 ± 0.18g	395.24 ± 0.39bc
*F. antarctica*	0.83 ± 0.01e	3.71 ± 0.07g	94.22 ± 0.07a	1.24 ± 0.01h	399.18 ± 0.06a
*F. endoxantha*	1.19 ± 0.03e	7.76 ± 0.33e	79.84 ± 0.58f	11.21 ± 0.55b	361.09 ± 1.43f
*G. gargal*	1.79 ± 0.09d	5.96 ± 0.49f	88.59 ± 0.53c	3.66 ± 0.28f	394.33 ± 1.12c
*L. nuda*	0.84 ± 0.02e	22.29 ± 0.44a	68.29 ± 0.66hi	8.58 ± 0.53c	369.84 ± 1.58e
*R. patagonica*	2.51 ± 0.10c	19.68 ± 0.64b	69.34 ± 0.53h	8.47 ± 0.25c	378.67 ± 0.71d
Homoscedasticity ^1^	0.027	0.288	0.036	0.256	0.199
1-way ANOVA ^2^	<0.001	<0.001	<0.001	<0.001	<0.001

^1^ Homoscedasticity among mushroom samples was tested by the Levene test: homoscedasticity, *p* > 0.05; heteroscedasticity, *p* < 0.05. ^2^ *p* < 0.05 indicates that the mean value of at least one component differs from the others (in this case, multiple comparison tests were performed). *p-*Value (*n* = 81). For each mushroom sample, means within a column with different letters differ significantly (*p* < 0.05).

**Table 2:** Sugars composition (g/100 g on dry weight basis) of the studied wild mushrooms (mean ± SD).

Species	Rhamnose	Fructose	Mannitol	Trehalosa	Sucrose	Total Sugars
*A. vitellinus*	0.58 ± 0.07c	0.67 ± 0.03c	1.44 ± 0.10d	3.38 ± 0.04d	nd	6.07 ± 0.24f
*H. dusenii*	nd	nd	2.57 ± 0.12c	10.26 ± 0.01a	1.25 ± 0.15	14.08 ± 0.28c
*C. magellanicus*	nd	nd	3.92 ± 0.16b	5.00 ± 0.03c	nd	8.92 ± 0.14e
*C. hariotii*	nd	nd	0.81 ± 0.01e	3.21 ± 0.02e	nd	4.02 ± 0.03g
*F. antarctica*	13.18 ± 0.32a	11.65 ± 0.37a	1.26 ± 0.20d	2.59 ± 0.09f	nd	28.68 ± 0.58a
*F. endoxantha*	6.09 ± 0.68b	9.92 ± 0.01b	nd	2.04 ± 0.02g	nd	18.05 ± 0.65b
*G. gargal*	nd	0.15 ± 0.02e	0.47 ± 0.11f	1.15 ± 0.07h	nd	1.77 ± 0.16h
*L. nuda*	nd	0.29 ± 0.02d	3.64 ± 0.34b	6.23 ± 0.17b	nd	10.16 ± 0.49d
*R. patagonica*	nd	0.29 ± 0.03d	9.12 ± 0.32a	nd	nd	9.41 ± 0.35e
Homoscedasticity ^1^	0.012	0.009	0.175	0.054	0.005	0.317
1-way ANOVA ^2^	<0.001	<0.001	<0.001	<0.001	<0.001	<0.001

^1^ Homoscedasticity among mushroom samples was tested by the Levene test: homoscedasticity, *p* > 0.05; heteroscedasticity, *p* < 0.05. ^2^ *p* < 0.05 indicates that the mean value of at least one component differs from the others (in this case, multiple comparison tests were performed). *p*-Value (*n* = 81). For each mushroom sample, means within a column with different letters differ significantly (*p* < 0.05). nd = not detected.

**Table 3:** Fatty acids composition (relative percentage) of the studied wild mushrooms (mean ± SD).

	*A. vitellinus*	*H. dusenii*	*C. magellanicus*	*C.hariotii*	*F. antarctica*	*F. endoxantha*	*G. gargal*	*L. nuda*	*R. patagonica*	Homoscedasticity ^1^	1-Way ANOVA ^2^
C6:0	0.06 ± 0.01c	0.55 ± 0.06a	0.25 ± 0.01b	0.06 ± 0.01c	0.18 ± 0.01b	0.20 ± 0.02b	0.07 ± 0.01c	0.20 ± 0.01b	0.081 ± 0.001c	0.188	<0.001
C8:0	0.04 ± 0.01cd	0.12 ± 0.01b	0.05 ± 0.01c	0.11 ± 0.02b	0.10 ± 0.01b	0.20 ± 0.02a	0.05 ± 0.00c	0.06 ± 0.01c	0.03 ± 0.01d	0.516	<0.001
C10:0	0.031 ± 0.001d	0.11 ± 0.01c	0.029 ± 0.001d	0.09 ± 0.01c	0.17 ± 0.03b	0.42 ± 0.04a	0.029 ± 0.001d	0.041 ± 0.001d	0.019 ± 0.001d	0.057	<0.001
C12:0	0.039 ± 0.001e	0.11 ± 0.01d	0.049 ± 0.001e	0.19 ± 0.01c	0.22 ± 0.04b	0.55 ± 0.02a	0.091 ± 0.001d	0.090 ± 0.001d	0.031 ± 0.001e	0.049	<0.001
C14:0	0.33 ± 0.05f	0.60 ± 0.01cd	0.22 ± 0.03f	0.66 ± 0.04c	1.57 ± 0.07b	2.43 ± 0.03a	0.25 ± 0.04f	0.44 ± 0.02e	0.53 ± 0.08de	0.122	<0.001
C14:1	0.04 ± 0.01c	0.051 ± 0.001c	0.011 ± 0.001d	0.05 ± 0.01c	0.10 ± 0.01b	0.15 ± 0.01a	0.13 ± 0.01ab	nd	nd	0.038	<0.001
C15:0	0.75 ± 0.06b	0.57 ± 0.01c	0.57 ± 0.03c	0.21 ± 0.02e	0.57 ± 0.04c	0.57 ± 0.01c	0.38 ± 0.04d	0.26 ± 0.03e	1.08 ± 0.08a	0.051	<0.001
C16:0	18.25 ± 0.86c	14.46 ± 0.28e	19.21 ± 0.70b	18.23 ± 0.24c	16.62 ± 0.79d	20.17 ± 0.12a	17.44 ± 0.46cd	12.45 ± 0.03g	13.44 ± 0.42f	0.297	<0.001
C16:1	1.45 ± 0.16b	0.43 ± 0.01f	0.38 ± 0.02fg	0.70 ± 0.04d	1.02 ± 0.08c	1.84 ± 0.01a	0.61 ± 0.01d	0.46 ± 0.02f	0.28 ± 0.08g	0.060	<0.001
C17:0	0.13 ± 0.01e	0.22 ± 0.01de	0.10 ± 0.01e	0.34 ± 0.03cd	0.68 ± 0.08b	0.39 ± 0.04c	0.13 ± 0.01e	0.15 ± 0.01e	1.16 ± 0.08a	0.012	<0.001
C18:0	3.69 ± 0.03c	3.95 ± 0.18c	3.30 ± 0.07d	3.72 ± 0.20c	7.37 ± 0.04a	4.52 ± 0.40b	7.31 ± 0.06a	1.95 ± 0.04e	3.84 ± 0.01c	0.032	<0.001
C18:1n9	52.08 ± 0.45a	33.73 ± 2.03c	9.04 ± 0.15gf	7.93 ± 0.80f	26.63 ± 0.02e	23.53 ± 0.01f	45.28 ± 0.03b	9.81 ± 0.22g	29.58 ± 0.67d	0.038	<0.001
C18:2n6	19.28 ± 0.60g	42.69 ± 1.69d	63.75 ± 0.69b	34.68 ± 0.29e	42.97 ± 1.13d	43.63 ± 0.85d	26.55 ± 0.03f	71.32 ± 0.24a	45.35 ± 0.69c	0.352	<0.001
C18:3n3	2.02 ± 0.16b	0.10 ± 0.01d	0.10 ± 0.01d	11.56 ± 0.40a	0.24 ± 0.06cd	0.13 ± 0.03cd	0.25 ± 0.01cd	0.39 ± 0.01c	0.07 ± 0.01d	0.019	<0.001
C20:0	0.29 ± 0.01c	0.07 ± 0.01f	0.22 ± 0.01d	2.70 ± 0.08a	0.16 ± 0.02de	0.10 ± 0.01ef	0.15 ± 0.01e	0.11 ± 0.01ef	0.59 ± 0.07b	0.049	<0.001
C20:1	0.17 ± 0.01b	0.10 ± 0.03c	0.06 ± 0.01d	0.07 ± 0.01d	0.10 ± 0.01c	0.07 ± 0.01d	0.16 ± 0.02b	nd	0.42 ± 0.01a	0.301	<0.001
C20:2	0.031 ± 0.001f	0.042 ± 0.001ef	0.09 ± 0.01d	1.08 ± 0.04a	0.041 ± 0.001ef	0.08 ± 0.01de	0.052 ± 0.001de	0.14 ± 0.01c	0.59 ± 0.04b	0.184	<0.001
C20:3n3 + C21:0	0.042 ± 0.001c	0.15 ± 0.03abc	0.34 ± 0.04a	0.25 ± 0.02abc	0.071 ± 0.001bc	0.23 ± 0.05abc	0.041 ± 0.001c	0.29 ± 0.01abc	0.31 ± 0.04ab	0.055	<0.001
C20:5n3	0.043 ± 0.002d	0.17 ± 0.04b	0.52 ± 0.05a	nd	0.051 ± 0.001d	nd	0.09 ± 0.01c	0.55 ± 0.02a	nd	0.074	<0.001
C22:0	0.45 ± 0.03d	0.17 ± 0.01g	0.91 ± 0.09c	3.84 ± 0.05a	0.38 ± 0.07e	0.25 ± 0.01f	0.32 ± 0.05ef	0.29 ± 0.05f	1.01 ± 0.02b	0.129	<0.001
C22:1n9	0.08 ± 0.01b	0.062 ± 0.001bc	0.11 ± 0.01b	0.21 ± 0.01a	nd	0.051 ± 0.002c	nd	0.21 ± 0.01a	0.091 ± 0.005b	0.040	<0.001
C23:0	0.062 ± 0.001b	nd	0.043 ± 0.001c	nd	0.041 ± 0.004c	0.028 ± 0.001c	nd	nd	0.45 ± 0.03a	0.021	<0.001
C24:0	0.55 ± 0.03c	0.46 ± 0.02c	0.17 ± 0.02e	5.71 ± 0.22a	0.52 ± 0.02c	0.32 ± 0.03d	0.53 ± 0.04c	0.54 ± 0.08c	0.94 ± 0.03b	0.041	<0.001
C24:1	0.09 ± 0.01e	1.07 ± 0.05b	0.49 ± 0.02c	7.61 ± 0.13a	0.21 ± 0.02de	0.13 ± 0.02de	0.09 ± 0.01e	0.23 ± 0.02d	0.11 ± 0.05de	0.076	<0.001
Total SFA	24.67 ± 1.01e	21.40 ± 0.03g	25.12 ± 0.71e	35.85 ± 0.47a	28.58 ± 0.97c	30.16 ± 0.51b	26.75 ± 0.41d	16.60 ± 0.03h	23.20 ± 1.08f	0.301	<0.001
Total MUFA	53.92 ± 0.29a	35.45 ± 1.84c	10.09 ± 0.10h	16.58 ± 1.29g	28.06 ± 0.10e	25.76 ± 0.02f	46.27 ± 0.05b	10.71 ± 0.28h	30.47 ± 0.67d	0.044	<0.001
Total PUFA	21.42 ± 0.72f	43.16 ± 1.88d	64.79 ± 0.81b	47.57 ± 0.81c	43.37 ± 1.07d	44.08 ± 0.53d	26.98 ± 0.36e	72.69 ± 0.25a	46.33 ± 0.40c	0.341	<0.001

(C6:0) caproic acid; (C8:0) caprylic acid; (C10:0) capric acid; (C12:0) lauric acid; (C14:0) myristic acid; (C14:1) myristoleic acid; (C15:0) pentadecanoic acid; (C16:0) palmitic acid; (C16:1) palmitoleic acid; (C17:0) heptadecanoic acid; (C18:0) stearic acid; (C18:1n9) oleic acid; (C18:2n6) linoleic acid; (C18:3n6) γ-linolenic acid; (C18:3n3) α-linolenic acid; (C20:1) cis-11-eicosaenoic acid; (C20:0) arachidic acid; (C20:2) cis-11,14-eicosadienoic acid; (C20:3n3 + C21:0) cis-11,14,17-eicosatrienoic acid + heneicosanoic acid; (C20:4n6) arachidonic acid; (C20:5n3) cis-5,8,11,14,17-eicosapentaenoic acid; (C21:0) heneicosanoic acid; (C22:0) behenic acid; (C22:1n9) erucic acid; (C23:0) tricosanoic acid; (C24:0) lignoceric acid; (C24:1) nervonic acid; (SFA) Saturated Fatty Acids; (MUFA) Monounsaturated Fatty Acids; (PUFA) Polyunsaturated Fatty Acids. ^1^ Homoscedasticity among mushroom samples was tested by the Levene test: homoscedasticity, *p* > 0.05; heteroscedasticity, *p* < 0.05. ^2^ *p* < 0.05 indicates that the mean value of at least one component differs from the others (in this case, multiple comparison tests were performed). *p*-Value (*n* = 81). For each mushroom sample, means within a column with different letters differ significantly (*p* < 0.05). nd = not detected.

**Table 4:** Organic acids composition (g/100 g of dry weight) of the studied wild mushrooms (mean ± SD).

Species	Oxalic Acid	Quinic Acid	Malic Acid	Citric Acid	Fumaric Acid	Total Identified Organic Acids
*A. vitellinus*	0.18 ± 0.01h	nd	nd	nd	0.03 ± 0.01h	0.21 ± 0.01h
*H. dusenii*	0.54 ± 0.01c	nd	nd	nd	0.31 ± 0.01d	0.85 ± 0.01f
*C. magellanicus*	0.38 ± 0.01d	nd	2.33 ± 0.04a	7.73 ± 0.03a	1.59 ± 0.01a	12.03 ± 0.01b
*C. hariotii*	0.27 ± 0.01e	nd	0.35 ± 0.01e	nd	0.06 ± .001g	0.68 ± 0.01g
*F. antarctica*	0.24 ± 0.01f	0.08 ± 0.01b	1.51 ± 0.07c	nd	0.51 ± 0.03c	2.34 ± 0.07d
*F. endoxantha*	0.10 ± 0.01i	nd	1.01 ± 0.01d	nd	0.30 ± 0.01d	1.41 ± 0.01e
*G. gargal*	0.22 ± 0.01g	nd	1.91 ± 0.01b	nd	0.19 ± 0.01f	2.32 ± 0.01d
*L. nuda*	2.22 ± 0.02b	26.41 ± 0.23a	nd	nd	0.24 ± 0.01e	28.87 ± 0.20a
*R. patagonica*	2.53 ± 0.02a	nd	nd	7.48 ± 0.04b	0.53 ± 0.01b	10.54 ± 0.01c
Homoscedasticity ^1^	0.036	0.005	0.006	0.006	0.122	0.016
1-way ANOVA ^2^	<0.001	<0.001	<0.001	<0.001	<0.001	<0.001

^1^ Homoscedasticity among mushroom samples was tested by the Levene test: homoscedasticity, *p* > 0.05; heteroscedasticity, *p* < 0.05. ^2^ *p* < 0.05 indicates that the mean value of at least one component differs from the others (in this case, multiple comparison tests were performed). *p*-Value (*n* = 81). For each mushroom sample, means within a column with different letters differ significantly (*p* < 0.05). nd = not detected.

**Table 5:** Composition in tocopherols (µg/100 g of dry weight) of the studied wild mushrooms (mean ± SD).

Species	α-Tocopherol	β-Tocopherol	Total Tocopherols
*A. vitellinus*	8.09 ± 0.42b	nd	8.09 ± 0.42e
*H. dusenii*	nd	nd	nd
*C. magellanicus*	30.65 ± 0.84a	59.03 ± 0.98a	89.68 ± 0.14a
*C. hariotii*	2.98 ± 0.01d	nd	2.98 ± 0.01f
*F. antarctica*	3.08 ± 0.14d	nd	3.08 ± 0.14f
*F. endoxantha*	5.13 ± 0.28c	29.88 ± 0.70c	35.01 ± 0.98c
*G. gargal*	2.99 ± 0.28d	12.36 ± 0.85d	15.35 ± 1.13d
*L. nuda*	1.99 ± 0.28e	36.25 ± 0.98b	38.24 ± 0.70b
*R. patagonica*	nd	nd	nd
Homoscedasticity ^1^	0.069	0.048	0.058
1-way ANOVA ^2^	<0.001	<0.001	<0.001

^1^ Homoscedasticity among mushroom samples was tested by the Levene test: homoscedasticity, *p* > 0.05; heteroscedasticity, *p* < 0.05. ^2^ *p* < 0.05 indicates that the mean value of at least one component differs from the others (in this case, multiple comparison tests were performed). *p-*Value (*n* = 81). For each mushroom sample, means within a column with different letters differ significantly (*p* < 0.05). nd = not detected.

**Table 6:** Composition in phenolic compounds of the studied wild mushrooms (µg/100g dw) (mean ± SD).

Species	Gallic Acid	*p*-Hydroxybenzoic Acid	*p*-Coumaric Acid	Total	Cinnamic Acid
*A. vitellinus*	1.26 ± 0.01c	tr	nd	1.26 ± 0.01e	nd
*H. dusenii*	nd	tr	nd	nd	nd
*C. magellanicus*	nd	nd	nd	nd	nd
*C. hariotii*	1.48 ± 0.07c	nd	nd	1.48 ± 0.07de	nd
*F. antarctica*	3.14 ± 0.05b	6.71 ± 0.25b	nd	9.85 ± 0.09b	nd
*F. endoxhanta*	4.59 ± 0.02a	nd	nd	4.59 ± 0.02c	nd
*G. gargal*	1.70 ± 0.01c	tr	nd	1.70 ± 0.01d	nd
*L. nuda*	nd	nd	nd	nd	nd
*R. patagonica*	4.56 ± 0.06a	126.42 ± 2.16a	3.41 ± 0.01	134.39 ± 2.10a	3.10 ± 0.01
Homoscedasticity ^1^	0.008	0.008	0.007	0.013	0.007
1-way ANOVA ^2^	<0.001	<0.001	<0.001	<0.001	<0.001

^1^ Homoscedasticity among mushroom samples was tested by the Levene test: homoscedasticity, *p* > 0.05; heteroscedasticity, *p* < 0.05. ^2^ *p* < 0.05 indicates that the mean value of at least one component differs from the others (in this case, multiple comparison tests were performed). *p*-Value (*n* = 81). For each mushroom sample, means within a column with different letters differ significantly (*p* < 0.05). nd = not detected.

**Table 7:** Total phenolics and in vitro antioxidant properties (EC_50_ values) of the studied wild mushrooms (mean ± SD).

Species	Phenolics (mg GAE/g extract)	DPPH Scavenging Activity (mg/mL)	Reducing Power (mg/mL)	β-Carotene Bleaching Inhibition (mg/mL)	TBARS Inhibition (mg/mL)
*A*. *vitellinus*	40.60 ± 1.82b	17.05 ± 0.34b	0.82 ± 0.01f	2.85 ± 0.18c	0.38 ± 0.02de
*H*. *dusenii*	16.40 ± 0.49e	17.88 ± 0.52b	1.24 ± 0.02e	2.31 ± 0.10d	1.33 ± 0.15b
*C*. *magellanicus*	9.86 ± 0.46f	15.72 ± 0.29c	3.77 ± 0.03a	1.05 ± 0.07e	0.85 ± 0.03c
*C*. *hariotii*	8.48 ± 0.38g	19.24 ± 0.74a	2.17 ± 0.03c	3.42 ± 0.11b	0.14 ± 0.01ef
*F*. *antarctica*	7.82 ± 0.19g	13.78 ± 0.18d	2.46 ± 0.03b	2.94 ± 0.08c	0.95 ± 0.04c
*F*. *endoxantha*	33.56 ± 0.84c	1.54 ± 0.02g	0.79 ± 0.01g	2.23 ± 0.07d	0.44 ± 0.06d
*G*. *gargal*	9.77 ± 0.23f	12.17 ± 0.05e	1.97 ± 0.03d	3.31 ± 0.16b	0.23 ± 0.01def
*L*. *nuda*	27.34 ± 0.63d	2.16 ± 0.13f	0.75 ± 0.01h	9.13 ± 0.23a	6.40 ± 0.14a
*R*. *patagonica*	50.82 ± 0.56a	0.77 ± 0.04h	0.17 ± 0.01i	0.61 ± 0.05f	0.06 ± 0.01f
Homoscedasticity ^1^	<0.001	<0.001	<0.001	<0.001	<0.001
1-way ANOVA ^2^	<0.001	<0.001	<0.001	<0.001	<0.001

^1^ Homoscedasticity among mushroom samples was tested by the Levene test: homoscedasticity, *p* > 0.05; heteroscedasticity, *p* < 0.05. ^2^ *p* < 0.05 indicates that the mean value of at least one component differs from the others (in this case, multiple comparison tests were performed). *p*-Value (*n* = 81). For each mushroom sample, means within a column with different letters differ significantly (*p* < 0.05).
